# High‐throughput discovery of functional disordered regions

**DOI:** 10.15252/msb.20188377

**Published:** 2018-05-22

**Authors:** Muhammad Ali, Ylva Ivarsson

**Affiliations:** ^1^ Department of Chemistry – BMC Uppsala University Uppsala Sweden

**Keywords:** Genome-Scale & Integrative Biology, Structural Biology, Transcription

## Abstract

Partially or fully intrinsically disordered proteins are widespread in eukaryotic proteomes and play important biological functions. With the recognition that well defined protein structure is not a fundamental requirement for function come novel challenges, such as assigning function to disordered regions. In their recent work, Babu and colleagues (Ravarani *et al*, 2018) took on this challenge by developing IDR‐Screen, a robust high‐throughput approach for identifying functions of disordered regions.

Over one‐third of the human proteome is intrinsically disordered and lacks stable secondary and tertiary structure (Tompa *et al*, 2014). The disordered regions contain a large proportion of the interaction and regulatory sites that are necessary for cell function and signaling, often in the form of short linear motifs. These short linear motifs are typically composed of 3–12 amino acid residues, of which a subset contributes to the protein–protein interactions. Regions that are intrinsically disordered can be robustly predicted based on amino acid composition as they are enriched in small, polar, and charged amino acids. It is however much more challenging to uncover the functionalities that reside within them. Embedded interaction sites can be found by methods such as proteomic peptide phage display (Davey *et al*, 2017), where disordered regions of a given proteome are displayed on the surface of phage particles that are then used in panning experiments against immobilized bait proteins. However, such experiments do not inform on the functional effects of the interactions. How will we then be able to assign functionalities to disordered regions? The task is daunting, as it has been estimated that there are up to 1 million motifs in disordered regions (Tompa *et al*, 2014).

To tackle this challenge, Ravarani *et al* (2018) developed a high‐throughput method, the intrinsically disordered region (IDR)‐Screen, that analyzes whether or not a disordered region can serve as a transactivation domain (TAD). Transactivation domain sequences function as binding sites for coactivators with varying structures, leading to the recruitment of the transcriptional machinery and the activation of transcription (Ptashne & Gann, 1997). These functional elements typically comprise <20 amino acids, are found in disordered regions, and fold to amphipathic helices upon binding (Sigler, 1988; Uesugi *et al*, 1997). Despite the large and growing number of identified TADs, it remains challenging to predict them using bioinformatics approaches due to their low sequence conservation. Their experimental discovery has been hampered by a lack of suitable high‐throughput methods. The IDR‐Screen is thus a welcome addition to the toolbox (Fig 1).

**Figure 1:**
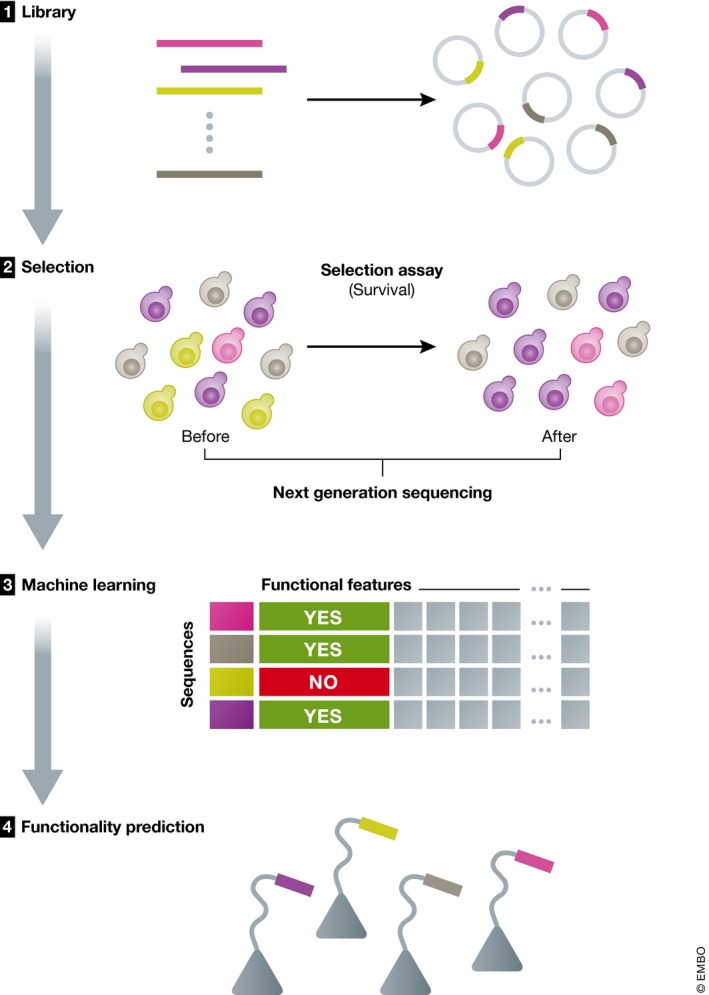
Summary of IDR‐Screen IDR‐Screen involves assembling a library of random or designed sequences and inserting it into the genome as a part of a protein used for selection (in this case, the selection is for heat‐shock survival). The library is screened to discover functional vs. non‐functional sequences. The dataset of experimentally validated functional and non‐functional sequences is then analyzed using machine learning, and features that can discriminate functional and non‐functional sequences are extracted.

The IDR‐Screen approach is based on a heat‐shock factor 1 (HSF1) transcription‐factor assay. HSF1 is required for yeast to sustain growth at elevated temperatures, and deletion of its C‐terminal TAD results in cell death at 37°C (Erkine & Gross, 2003). Ravarani *et al* took advantage of this property by creating cell populations in which the natural HSF1 TAD sequence was replaced by libraries of randomized sequences. Cells containing HSF1 linked to sequences that recruit coactivators survived incubation at 37°C, while those with non‐functional sequences did not. Sequencing of the cell populations before and after selection at 37°C generated information on both functional and non‐functional sequences, which is a strength of the method. However, a limitation is its screening capacity: Although more than 100,000 random sequences were screened for function, this only represents a very tiny fraction of the theoretical sequence space of such a library. Therefore, the sampling is sparse and the method might be better suited for specifically designed libraries.

More than 700 random sequences, corresponding to about 1% of the screened number, were found to function as TADs, which suggests that the requirements on sequences to function as TADs are rather relaxed. This is consistent with earlier findings by Ma and Ptashne (1987). Compared to non‐functional sequences, the identified TAD sequences are enriched in acidic and hydrophobic residues and depleted of basic residues. Subsequently, machine learning was used to capture the features that define TADs, using the generated information on functional and non‐functional sequences, revealing them as the presence of multiple degenerate “mini‐motifs” of negatively charged and aromatic amino acids, and the absence of positively charged residues. The importance of acidic residues for preventing hydrophobic motifs from driving collapse was recently elucidated by mutational scanning in a related study (Staller *et al*, 2018). The IDR‐Screen approach was further showcased by a mutational analysis of 13 naturally occurring TADs from various organisms. The functions of these sequences were found to be generally robust to mutation, except for when basic residues were introduced or key hydrophobic residues mutated. Finally, the authors combined the results from the distinct screens and generated an optimized predictor for functional TADs. This however raises some concerns, as the results appear to be biased toward tryptophan‐rich, overly hydrophobic sequences. Such bias has previously been shown to hamper predictions of binding motifs in the proteome based on peptide phage display results (Luck & Travé, 2011). The combined use of the results from the IDR‐Screen of random libraries and the library designed based on natural occurring TADs may make the predictions more reliable.

The relaxed requirements on TADs and their robustness against mutation raise intriguing questions about promiscuity and selectivity. Do TADs with generic sequences interact with certain subgroups of coactivators? Do they engage in “fuzzy complexes” as suggested by Ravarani *et al* (2018)? How is specificity achieved in the cellular system? Further studies will be necessary to answer these questions. Taken together, IDR‐Screen is a powerful method for identifying function of disordered regions in a given system. It can be further developed to explore other functionalities of intrinsically disordered regions, and it will be a valuable approach for shedding light on this largely underexplored part of the proteome.
